# Database for the *ampC* alleles in *Acinetobacter baumannii*

**DOI:** 10.1371/journal.pone.0176695

**Published:** 2017-05-01

**Authors:** Nabil Karah, Keith A. Jolley, Ruth M. Hall, Bernt Eric Uhlin

**Affiliations:** 1 The Laboratory for Molecular Infection Medicine Sweden (MIMS) and Department of Molecular Biology, Umeå University, Umeå, Sweden; 2 Umeå Centre for Microbial Research, Umeå University, Umeå, Sweden; 3 Department of Zoology, University of Oxford, Oxford, United Kingdom; 4 School of Life and Environmental Sciences, University of Sydney, Sydney, Australia; Tianjin University, CHINA

## Abstract

*Acinetobacter baumannii* is a troublesome opportunistic pathogen with a high capacity for clonal dissemination. We announce the establishment of a database for the *ampC* locus in *A*. *baumannii*, in which novel *ampC* alleles are differentiated based on the occurrence of ≥ 1 nucleotide change, regardless of whether it is silent or missense. The database is openly accessible at the pubmlst platform for *A*. *baumannii* (http://pubmlst.org/abaumannii/). Forty-eight distinctive alleles of the *ampC* locus have so far been identified and deposited in the database. Isolates from clonal complex 1 (CC1), according to the Pasteur multilocus sequence typing scheme, had a variety of the *ampC* locus alleles, including alleles 1, 3, 4, 5, 6, 7, 8, 13, 14, 17, and 18. On the other hand, isolates from CC2 had the *ampC* alleles 2, 3, 19, 20, 21, 22, 23, 24, 26, 27, 28, and 46. Allele 3 was characteristic for sequence types ST3 or ST32. The *ampC* alleles 10, 16, and 25 were characteristic for CC10, ST16, and CC25, respectively. Our study points out that novel gene databases, in which alleles are numbered based on differences in their nucleotide identities, should replace traditional records that use amino acid substitutions to define new alleles.

## Introduction

*Acinetobacter baumannii* is a clinically important pathogen responsible for a wide range of hospital-acquired infections [1]. The *ampC* gene of *A*. *baumannii* was cloned and sequenced for the first time in 2000 [2]. The gene, also called *bla*_ADC_ for *A**cinetobacter*-Derived Cephalosporinase, is intrinsic in *A*. *baumannii* and all other members of the *A**cinetobacter* *c**alcoaceticus-Acinetobacter* *b**aumannii* (*Acb*) complex [3, 4]. It is located in the chromosome between *folE*, encoding a GTP cyclohydrolase I enzyme, and an open reading frame encoding a hypothetical protein, as seen in the *A*. *baumannii* reference strain ATCC 17978-mff (GenBank accession number CP012004, locus tag ACX60_05710). Overexpression of *ampC*, due to the acquisition of a strong promoter located on an insertion sequence (IS) element, is the main mechanism of resistance to third-generation cephalosporins in *A*. *baumannii* [5]. With few exceptions, variation in the amino acid sequence of AmpC in *A*. *baumannii* usually does not affect the resistance spectrum [6, 7].

Some *A*. *baumannii* isolates were reported to carry a second copy of the *ampC* gene, located elsewhere in the chromosome [8, 9]. The additional copy was part of a DNA segment most likely derived from the chromosome of another *A*. *baumannii* strain. The segment was mobilized as part of Tn*6168*, a composite transposon made of two directly oriented copies of IS*Aba1* [8]. The *A*. *baumannii ampC* gene, together with an upstream IS*Our1*, was also detected in the genome of *Oligella urethralis*, leading to a cephalosporin resistance phenotype [10]. Interestingly, *A*. *baumannii* strain ACICU, from global clone 2 (GC2), was found to carry a 9 kb chromosomal segment, containing IS*Aba125*-*ampC*, which was derived from a GC1 isolate [11]. This finding indicated the occurrence of a replacement in the chromosome of ACICU, most likely mediated by a homologous recombination event [11]. Similarly, distinctive IS*Aba1*-associated *ampC* alleles were detected in the genome of GC1 isolates, once again highlighting the frequent occurrence of horizontal transfer of chromosomal DNA segments in *A*. *baumannii* [9, 12].

To track these imports, a clear numbering system of the *ampC* alleles is needed. Analysis of the *ampC* locus could also be a convenient method for exploring the molecular epidemiology of *A*. *baumannii*, taking into consideration that particular *ampC* alleles have been linked to certain clones of *A*. *baumannii* [9, 13]. This report aims to announce the establishment of a database for the *ampC* locus in *A*. *baumannii*.

## New database for the *ampC* locus in *A*. *baumannii*

The database is hosted and maintained at the pubmlst platform for *A*. *baumannii* (http://pubmlst.org/abaumannii/) sited at the University of Oxford [14]. The platform provides an open access to all the data and allows submissions of novel sequences. However, novel sequence must simultaneously be submitted and assigned accession numbers by the International Nucleotide Sequence Database Collaboration (INSDC) (http://www.insdc.org/). Sequences must be complete and meet the validation criteria of INSDC. *ampC* sequences with novel nucleotide identities (≥ 1 nucleotide substitution) will be numbered successively.

So far, we have identified, curated and numbered a total of 48 distinctive alleles of the *ampC* locus in a collection of 188 *A*. *baumannii* isolates by means of the online available whole genome sequence records (Table 1). The *ampC* alleles 1, 3, 4, 5, 6, 7, 8, 13, 14, 17, and 18 were carried by isolates that belong to clonal complex 1 (CC1), corresponding to GC1, according to the Pasteur scheme for multilocus sequence typing (https://pubmlst.org/abaumannii/). Isolates from CC2, corresponding to GC2, had the *ampC* alleles 2, 3, 19, 20, 21, 22, 23, 24, 26, 27, 28, and 46. Nonetheless, *ampC* allele 2 was also present in one isolate from ST215 (27, 2, 7, 2, 2, 1, 2), which was not closely related to CC2. Similarly, allele 19 was present in isolates of ST500 (3, 3, 2, 2, 28, 1, 5) or ST522 (3, 3, 89, 2, 28, 1, 5), which were also not related to CC2. Although it was present in few isolates from CC1 and CC2, allele 3 was mainly characteristic for ST3 (3, 3, 2, 2, 3, 1, 3) or ST32 (1, 1, 2, 2, 3, 4, 4). The *ampC* locus alleles 10 and 16 were characteristic for CC10 and ST16, respectively. Likewise, all isolates from CC25 had the *ampC* locus allele 25. Allele 39 was present in all the ST78 (25, 3, 6, 2, 28, 1, 29) isolates, but also in one isolate from ST241 (40, 3, 15, 2, 40, 4, 4).

**Table 1:** Numeration of the *ampC* gene alleles in *Acinetobacter baumannii*.

***ampC* allele**	**Isolate**	**Pasteur scheme multi locus sequence type**	**GenBank accession**	**PubMed IDentifier (PMID) / GenBank submission authors / other references**
1	AYE	ST1 (1, 1, 1, 1, 5, 1, 1)	NC_010410	16415984; [9]
	AB5075	ST1	CP008706; AHAH00000000	24865555; [9]
	A1	ST1	CP010781	25767221; [9]
	3208	ST1	FJ172370.5; FBWZ00000000	19364869; [9]
	D2	ST1	GQ406245.5; FBWY00000000	20375036; [9]
	A92	ST1	GQ406246.3; FBWV00000000	20375036; [9]
	A85 (intrinsic)	ST1	KC118540.6; FBXA00000000	24907141; [9]
	AB307-0294	ST1	CP001172	18931120; [9]
	AB0057 (intrinsic)	ST1	CP001182	18931120; [9]
	6772166 (intrinsic)	ST1	FBWX00000000	[9]
	RBH3 (intrinsic)	ST1	FBXD00000000	[9]
	AB056 (intrinsic)	ST1	ADGZ00000000	20530228; [9]
	AB059 (intrinsic)	ST1	ADHB00000000	20530228; [9]
	AB_908–13 (intrinsic)	ST1	AMHW00000000	23365658; [9]
	AB_909-02-7 (intrinsic)	ST1	AMHZ00000000	23365658; [9]
	TG19582	ST1	AMIV00000000	23365658; [9]
	Canada BC-1 (intrinsic)	ST1	AMSZ00000000	Harkins *et al*., unpublished; [9]
	Canada BC-5 (intrinsic)	ST1	AFDN00000000	Harkins *et al*., unpublished; [9]
	IS-58	ST1	AMGH00000000	Harkins *et al*., unpublished; [9]
	IS-235	ST1	AMEI00000000	Harkins *et al*., unpublished
	IS-251	ST1	AMEJ00000000	Harkins *et al*., unpublished
	NIPH 290	ST1	APRD00000000	Feldgarden *et al*., unpublished; [9]
	NIPH 527 (RUH875)	ST1	APQW00000000	Cerqueira *et al*., unpublished; [9]
	ANC 4097	ST1	APRF00000000	Cerqueira *et al*., unpublished; [9]
	Naval-83	ST20 (3, 1, 1, 1, 5, 1, 1)	AMFK00000000	Harkins *et al*., unpublished; [9]
2	A91	ST2 (2, 2, 2, 2, 2, 2, 2)	JN968483	22351684
	NIPH 2061	ST2	APOW00000000	24277043
	OIFC180	ST2	AMDQ00000000	Harkins *et al*., unpublished
	CI77	ST2	AVOC00000000	24503987
	MRY09-0642	ST2	BASA00000000	23868126
	ORAB01	ST2	CP015483	Adams *et al*., unpublished
	XH856	ST2	CP014541	Feng *et al*., unpublished
	YU-R612	ST2	CP014215	27139604
	XH386	ST2	CP010779	26981403
	NCGM 237	ST2	AP013357	24550340
	BJAB0868	ST2	CP003849	23826102
	BJAB07104	ST2	CP003846	23826102
	MDR-ZJ06	ST2	CP001937	21788470
	TCDC-AB0715	ST2	CP002522.2	21398540
	ABNIH2	ST2	AFTA00000000	21825119
	AB210	ST2	AEOX00000000	21565804
	Naval-17	ST2	AFDO00000000	Harkins *et al*., unpublished
	Ab11111	ST2	AKAQ00000000	Murphy *et al*., unpublished
	ZWS1122	ST2	AMGR00000000	23209232
	ZWS1219	ST2	AMGS00000000	23209232
	Naval-113	ST2	AMZU00000000	Harkins *et al*., unpublished
	XH857	ST215 (27, 2, 7, 2, 2, 1, 2)	CP014540	Feng *et al*., unpublished
3	A085	ST3 (3, 3, 2, 2, 3, 1, 3)	KP881239	26824943
	AB4456	ST3	LREF00000000	Arivett *et al*., unpublished
	AB3560	ST3	LRDV00000000	Arivett *et al*., unpublished
	AB4857	ST3	AHAG00000000	22374953
	OIFC137	ST3	AFDK00000000	Harkins *et al*., unpublished
	OIFC109	ST3	ALAL00000000	Harkins *et al*., unpublished
	IS-123	ST3	ALII00000000	Harkins *et al*., unpublished
	Naval-81	ST3	AFDB00000000	Harkins *et al*., unpublished
	Naval-13	ST3	AMDR00000000	Harkins *et al*., unpublished
	WC-A-694	ST3	AMTA00000000	Harkins *et al*., unpublished
	OIFC032	ST32 (1, 1, 2, 2, 3, 4, 4)	AFCZ00000000	Harkins *et al*., unpublished
	OIFC087	ST32	AMFS00000000	Harkins *et al*., unpublished
	OIFC099	ST32	AMFT00000000	Harkins *et al*., unpublished
	1525283	ST32	JEXR00000000	Harris *et al*., unpublished
	781407	ST32	JEZS00000000	Harris *et al*., unpublished
	ABBL013	ST32	LLCT00000000	26699703
	OIFC074	ST19 (1, 2, 1, 1, 5, 1, 1)	AMDE00000000	Harkins *et al*., unpublished; [9]
	Naval-21	ST19	AMSY00000000	Harkins *et al*., unpublished; [9]
	1999BJAB11	ST2	JSDB00000000	25487793
	IS-143	ST414 (2, 2, 2, 2, 2, 37, 2)	AMGE00000000	Harkins *et al*., unpublished
4	D15	ST1	FBXJ00000000	[9]
	D13	ST1	FBXI00000000	[9]
5	G7	ST1	FBXF00000000	[9]
6	AB058	ST20	ADHA00000000	20530228; [9]
7^a^	A388	ST1	JQ684178; FBXE00000000	22915466; [9]
	A100	ST1	KP881241	26824943
8^a^	A85 (acquired)	ST1	KC118540.6; FBXA00000000	24907141
	AB0057 (acquired)	ST1	CP001182	18931120; [9]
	6772166 (acquired)	ST1	FBWX00000000	[9]
	RBH3 (acquired)	ST1	FBXD00000000	[9]
	AB056 (acquired)	ST1	ADGZ00000000	20530228; [9]
	AB059 (acquired)	ST1	ADHB00000000	20530228; [9]
	AB_908–13 (acquired)	ST1	AMHW00000000	23365658; [9]
	AB_909-02-7 (acquired)	ST1	AMHZ00000000	23365658; [9]
	Canada BC-1 (acquired)	ST1	AMSZ00000000	Harkins *et al*., unpublished; [9]
	Canada BC-5 (acquired)	ST1	AFDN00000000	Harkins *et al*., unpublished; [9]
9	NIPH 190	ST9 (3, 1, 5, 3, 6, 1, 3)	APPL00000000	24277043
10	T214	ST10 (1, 3, 2, 1, 4, 4, 4)	JRTZ00000000	Kamolvit *et al*., unpublished
	NIPH 335	ST10	APQX00000000	Cerqueira *et al*., unpublished
	OIFC098	ST10	AMDF00000000	Harkins *et al*., unpublished
	466760	ST10	JEXB00000000	Harris *et al*., unpublished
	50595	ST10	JEXP00000000	Harris *et al*., unpublished
	3390	ST10	JFER00000000	Harris *et al*., unpublished
	1262761–105	ST10	JMOJ00000000	Harris *et al*., unpublished
	Ab04-mff	ST10	CP012006	26170289
	A078	ST23 (1, 3, 10, 1, 4, 4, 4)	KP881236	26824943
	BJAB0715	ST23	CP003847	23826102
	XH858	ST23	CP014528	Feng *et al*., unpublished
11	NIPH 329	ST11 (1, 2, 6, 2, 3, 4, 4)	APQY00000000	24277043
12	NIPH 615	ST12 (3, 5, 7, 1, 7, 2, 6)	APOV00000000	24277043
13	A076	ST1	KP881235	26824943
14	A082	ST1	KP881238	26824943
15^b^	NIPH 1734 (LUH 8406)	ST15 (6, 6, 8, 2, 3, 5, 4)	APOX00000000	24277043
16	UMB002	ST16 (7, 7, 2, 2, 8, 4, 4)	AEPL00000000	21639920
	1043794	ST16	JEYX00000000	Harris *et al*., unpublished
	972082	ST16	JFAA00000000	Harris *et al*., unpublished
	232184	ST16	JEYI00000000	Harris *et al*., unpublished
	268680	ST16	JEYN00000000	Harris *et al*., unpublished
	655378	ST16	JFCE00000000.2	Harris *et al*., unpublished
	1064293_45	ST16	JFDS00000000	Harris *et al*., unpublished
17^c^	D36	ST81 (1, 1, 1, 1, 5, 1, 2)	CP012952	26679588; [9]
18^c^	D81	ST1	FBXC00000000	[9]
	D78	ST1	FBXH00000000	[9]
19^c^	RUH 134 (A320)	ST2	JN247441	23788477
	NIPH 24	ST2	APOF00000000	Cerqueira *et al*., unpublished
	NIPH 528	ST2	APRB00000000	Cerqueira *et al*., unpublished
	OIFC338	ST2	AMFX00000000	Harkins *et al*., unpublished
	XH859	ST2	CP014539	Feng *et al*., unpublished
	AB1H8	ST2	ANNC00000000	23723398
	AB5711	ST2	AHAJ00000000	22374953
	472237–120	ST500 (3, 3, 2, 2, 28, 1, 5)	JFCW00000000	Harris *et al*., unpublished
	1188188	ST500	JFDV00000000	Harris *et al*., unpublished
	1271213	ST500	JFDX00000000	Harris *et al*., unpublished
	1237893	ST500	JFEA00000000	Harris *et al*., unpublished
	480175	ST500	JFEU00000000	Harris *et al*., unpublished
	1276470–86	ST500	JFXE00000000	Harris *et al*., unpublished
	1121032	ST500	JEZE00000000.2	Harris *et al*., unpublished
	940793	ST500	JMNW00000000	Harris *et al*., unpublished
	29280	ST522 (3, 3, 89, 2, 28, 1, 5)	JEZI00000000	Harris *et al*., unpublished
20	A072	ST2	KP881233	26824943
	XH860	ST2	CP014538	Feng *et al*., unpublished
	AC29	ST2	CP007535	26824943
	AC30	ST2	CP007577	26824943
	PKAB07	ST2	CP006963	24652977
21	J65	ST2	JQ867374	Wang, unpublished
22^d^	MDR_MMC4	ST2	AZNQ00000000	20609238
23	1656–2	ST2	CP001921	22038960
	DU202	ST2	AVGF00000000	24486871
24	TYTH-1	ST2	CP003856	23209228
	KBN10P02143	ST2	CP013924	27143492
25	OIFC143	ST25 (3, 3, 2, 4, 7, 2, 4)	AFDL00000000	Harkins *et al*., unpublished
	Naval-18	ST25	AFDA00000000	Harkins *et al*., unpublished
	NIPH 146	ST25	APOU00000000	Cerqueira *et al*., unpublished
	CI86	ST25	AVOB00000000	24503987
	CI79	ST25	AVOD00000000	24503987
	984213	ST25	JEVX00000000	Harris *et al*., unpublished
	1429530	ST25	JEWM00000000	Harris *et al*., unpublished
	NM3	ST25	JZBV00000000	23264451
	RUH 1486	ST25	JZBU00000000	26462752
	LUH 6220	ST25	JZBW00000000	26462752
	161/07	ST25	JZCA00000000	26462752
	4390	ST25	JZBY00000000	26462752
	LUH 7841	ST402 (3, 3, 2, 1, 7, 2, 4)	JZBX00000000	26462752
26	58452	ST2	JEZV00000000	Harris *et al*., unpublished
27	UH10007	ST2	AYGO00000000	24449752
28^d^	Naval-2	ST2	AMSX00000000	Harkins *et al*., unpublished
	TG15234	ST2	ASEW00000000	23365658
	TG15240	ST2	ASFB00000000	23365658
	1043903	ST2	JEYY00000000	Harris *et al*., unpublished
	17534	ST2	JEYQ00000000	Harris *et al*., unpublished
	1294217	ST2	JEWF01000000	Harris *et al*., unpublished
	1406750	ST2	JEWK00000000	Harris *et al*., unpublished
	724909	ST2	JEXF01000000	Harris *et al*., unpublished
	UMB001	ST2	AEPK00000000	21639920
	ABIsac_ColiS	ST2	CAKA00000000	23070160
29	NIPH 1669	ST3	APOQ00000000	24277043
30^e^	LAC-4	ST10	JICJ00000000	Cerqueira *et al*., unpublished
31	D46	ST25	KF030679.2	23788477
32	NIPH 60	ST34 (8, 1, 14, 3, 12, 1, 13)	APPM00000000	24277043
33	NIPH 67	ST35 (9, 3, 2, 2, 5, 4, 14)	APRA00000000	24277043
34	NIPH 80	ST37 (3, 2, 2, 2, 7, 1, 2)	APRE00000000	24277043
35	NIPH 201	ST38 (3, 2, 15, 6, 6, 4, 5)	APQV00000000	24277043
36	NIPH 601	ST40 (1, 2, 2, 2, 5, 1, 14)	APQZ00000000	24277043
37	J9	ST49 (3, 3, 6, 2, 3, 1, 5)	KF002790	23920428
38	ATCC 19606	ST52 (3, 2, 2, 7, 9, 1, 5)	APRG00000000	24277043
39	UH5207	ST78 (25, 3, 6, 2, 28, 1, 29)	AYFP00000000	24449752
	1096934	ST78	JEXM00000000	Harris *et al*., unpublished
	831240	ST78	JEYO00000000	Harris *et al*., unpublished
	855125	ST78	JMNT00000000	Harris *et al*., unpublished
	118362	ST241 (40, 3, 15, 2, 40, 4, 4)	JEWB00000000	Harris *et al*., unpublished
40	A099	ST85 (5, 2, 4, 1, 3, 3, 4)	KP881240	26824943
41	RBH2	ST111 (3, 3, 2, 2, 4, 8, 12)	KF030678	23788477
42	CIP 70.10	ST126 (3, 2, 7, 2, 7, 1, 3)	LN865143	Wibberg *et al*., unpublished
	233846	ST126	JMOG00000000	Harris *et al*., unpublished
	1419130	ST529 (3, 3, 7, 52, 7, 1, 4)	JEWL00000000	Harris *et al*., unpublished
43	AA-014	ST158 (41, 42, 13, 1, 5, 4, 14)	AMGA00000000	Harkins *et al*., unpublished
44	AB4A3	ST255 (3, 37, 2, 2, 42, 1, 14)	AOLU00000000	23723398
45	AB_TG2030	ST406 (1, 1, 1, 2, 65, 1, 5)	AMIJ00000000	Sahl *et al*., unpublished
46	16553_10	ST415 (2, 2, 2, 2, 68, 2, 2)	JHPF0000000	Harris *et al*., unpublished
47	1406589	ST521 (3, 88, 2, 2, 28, 1, 5)	JFYI00000000	Harris *et al*., unpublished
48	A074	ST636 (2, 1, 2, 2, 2, 1, 1)	KP881234	26824943

^a^ Indicates extra 15 nucleotides in the DNA sequence of alleles 7 and 8, which were previously designated as alleles 1 and 3, respectively [9]; The two alleles have different extra nucleotides.

^b^ Allele 15 was also detected in the genome of Oligella urethralis [2]

^c^ Alleles 17, 18, and 19 were previously designated as 1a,1b, and 2, respectively [9]

^d^ Indicates extra 3 nucleotides in the DNA sequence of alleles 22 and 28; The two alleles have the same extra nucleotides.

^e^ Indicates extra 9 nucleotides in the DNA sequence of allele 30.

These linkages demonstrate that sequence analysis of the *ampC* variants is probably a practical method to search for clinically significant clones of *A*. *baumannii*, as previously described for the intrinsic *bla*_OXA-51-like_ gene [15, 16] However, the frequent occurrence of inter-strain exchanges of chromosomal segments should be taken into consideration. Therefore, analysis of *ampC* to study the epidemiology of *A*. *baumannii* should be complemented by characterizing other loci or preferably be taken within the context of whole-genome sequence analysis.

## Updated list of the AmpC protein variants

In parallel, we revised and updated a previous collection of the AmpC variants (Table 2) [13]. As previously recommended, the AmpC variants were numbered according to the chronology of getting published and/or submitted to the INSDC databases. Numbers were preceded by a hyphen. When it was possible, numbers assigned by previous studies were retained. Accordingly, AmpC-1 was used to label the first AmpC protein variant reported in 2000 [2, 13]. The designation AmpC-72 (GenBank accession: AIL90389) was omitted since it showed 100% amino acid similarity to AmpC-70 (GenBank accession: KQG48886). Two variants with different amino acid sequences were designated as AmpC-57 (GenBank accessions: ADO51072 and AEZ36052). Subjectively, AmpC-57 was given to the variant detected in two *A*. *baumannii* isolates from East Africa [17]. New variants were defined, based on ≥ 1 amino acid substitution, and numbered under supervision of the INSDC curators. It is very important to re-emphasize that the AmpC variant numbers (Table 2) are not matching and not exchangeable with the *ampC* allele numbers (Table 1).

**Table 2:** Numeration of the AmpC protein variants encoded by *Acinetobacter baumannii*.

**AmpC protein variant**	**GenBank accession number**	**Size (amino acid)**	**NCBI reference sequence**	**Other previous designations**	**PubMed IDentifier (PMID) / GenBank submission authors**
AmpC-1 (ADC-1)	CAB77444	383	WP_004714775	ADC-NIPH 1362	10639377
AmpC-2 (ADC-2)	AAO43172	383	WP_004746565	ADC-NIPH 1734	12709319
AmpC-3	AAO59456	383	WP_063857798	−	12709319
AmpC-4	AAO59457	383	WP_063857801	−	12709319
AmpC-5	CAE00827	383	WP_038405930	−	15047547
AmpC-6	AAR13676	383	WP_017725267	−	14742218
AmpC-7	AAT70411	383	WP_063857816	−	15980372
AmpC-10	ABI18382	388	WP_063857786	−	Hujer *et al*., unpublished
AmpC-11	ADG46039	383	WP_001211205	−	20713667; 16415984
AmpC-12	CAK95249	383	WP_063857787	−	19029333
AmpC-13	CAK95248	383	WP_063857788	−	19029333
AmpC-14	CAK95247	383	WP_063857789	−	19029333
AmpC-15	CAK95246	383	WP_063857790	−	19029333
AmpC-16	CAK95245	383	WP_063857791	−	19029333
AmpC-17	CAK95244	383	WP_063857792	−	19029333
AmpC-18	CAK95243	383	WP_002118772	−	19029333
AmpC-19	CAK95242	383	WP_063857793	−	19029333
AmpC-20	CAK95241	383	WP_063857794	−	19029333
AmpC-21	CAK95240	383	WP_063857795	−	19029333
AmpC-22	CAK95239	383	WP_063857796	−	19029333
AmpC-23	CAK95238	383	WP_063857797	−	19029333
AmpC-24	CAK95237	383	−	ADC-19	Beceiro & Bou., unpublished
AmpC-25	ABK34773	383	WP_001211217	ADC-NIPH 528	18077141
AmpC-26	ADG46043	383	WP_001211238	ADC-NIPH 146	20713667
AmpC-29	ACC66195	383	−	−	Chiu *et al*., unpublished
AmpC-30	ADG46041	383	WP_001211218	ADC-NIPH 2061	20713667
AmpC-31	ADX04315	383	WP_001211223	−	22038960
AmpC-32	ENU68675	383	WP_004739487	ADC-NIPH 615	24277043
AmpC-38	ACC95873	383	WP_063857799	−	18765689
AmpC-39	ACC95874	383	WP_063857800	−	18765689
AmpC-41	ACN62070	383	WP_063857802	−	20368407
AmpC-42	ACN62071	383	WP_063857803	−	20368407
AmpC-43	ACN62072	383	WP_032055358	−	20368407
AmpC-44	ACN62073	383	WP_063857804	−	20368407
AmpC-50	ADG46038	383	WP_031965243	−	Rodriguez-Martinez *et al*., unpublished
AmpC-51	ADG46040	383	WP_063857805	−	20713667
AmpC-52	ADG46042	383	WP_001211232	−	20713667
AmpC-53	ADG46044	383	WP_063857806	−	20713667
AmpC-54	ADK35761	383	WP_063857807	−	20805394
AmpC-56	AEL30570	383	WP_031973850	−	21788456
AmpC-57	ADO51072	383	WP_001211226	−	24176550
AmpC-58	AFG25594	383	WP_063857808	−	Zhang, unpublished
AmpC-59	AFG25595	383	WP_063857809	−	Zhang, unpublished
AmpC-60	AFH53180	383	WP_063857810	−	Huang, unpublished
**AmpC protein variant**	**GenBank accession number**	**Size (amino acid)**	**NCBI reference sequence**	**Other previous designations**	**PubMed IDentifier (PMID) / GenBank submission authors**
AmpC-61	AFI56570	383	WP_033503051	−	Zhou, unpublished
AmpC-62	AFK24475	383	WP_063857811	−	Wang, unpublished
AmpC-63	AFM80040	383	WP_063857812	−	Zhang, unpublished
AmpC-65	AFP73417	385	−	−	Ling, unpublished
AmpC-66	AFP73418	383	−	−	Ling, unpublished
AmpC-67	AEZ36052	383	WP_063857814	ADC-57	Zhou, unpublished; 24619228
AmpC-68	AGL39360	383	WP_063857815	−	Lee *et al*., 2014 (as a poster); 25372683
AmpC-70	KQG48886	383	WP_017480710	ADC-72^a^	Ozer *et al*., unpublished
AmpC-73	ALA14808	383	WP_001211219	−	26824943
AmpC-74	ALA14809	383	WP_001211203	−	26824943
AmpC-75	ALA14810	383	WP_063857817	−	26824943
AmpC-76	ALA14811	383	WP_001211237	ADC-NIPH 335	26824943
AmpC-77	ALA14812	383	WP_063857818	−	26824943
AmpC-78	ALA14813	383	WP_057691006	−	26824943
AmpC-79	ALA14814	383	WP_001159760	−	26824943
AmpC-80	ALA14815	383	WP_029424536	−	26824943
AmpC-81	ALA14816	388	WP_059262723	−	26824943
AmpC-82	AOA49613	383	−	−	Saranathan *et al*., unpublished
AmpC-83	ANW47146	383	−	−	Kulkarni *et al*., unpublished
AmpC-84	ANW47149	383	−	−	Kulkarni *et al*., unpublished
AmpC-85	ANW47142	383	−	−	Kulkarni *et al*., unpublished
AmpC-86	ANW47143	383	−	−	Kulkarni *et al*., unpublished
AmpC-87	ANW47154	383	−	−	Kulkarni *et al*., unpublished
AmpC-88	ANW47135	383	−	−	Kulkarni *et al*., unpublished
AmpC-89	ANW47136	383	−	−	Kulkarni *et al*., unpublished
AmpC-90	ANW47147	383	−	−	Kulkarni *et al*., unpublished
AmpC-91	ANW47132	383	−	−	Kulkarni *et al*., unpublished
AmpC-92	ANW47134	383	−	−	Kulkarni *et al*., unpublished
AmpC-93	ANW47145	383	−	−	Kulkarni *et al*., unpublished
AmpC-94	ANW47137	383	−	−	Kulkarni *et al*., unpublished
AmpC-95	ANW47153	383	−	−	Kulkarni *et al*., unpublished
AmpC-96	ANW47150	388	−	−	Kulkarni *et al*., unpublished
AmpC-97	ANW47139	383	−	−	Kulkarni *et al*., unpublished
AmpC-98	ANW47138	383	−	−	Kulkarni *et al*., unpublished
AmpC-99	ANW47140	383	−	−	Kulkarni *et al*., unpublished
AmpC-100	ANW47141	385	−	−	Kulkarni *et al*., unpublished
AmpC-101	ANW47133	383	−	−	Kulkarni *et al*., unpublished
AmpC-102	ANW47148	383	−	−	Kulkarni *et al*., unpublished
AmpC-103	ANW69905	383	−	−	Kulkarni *et al*., unpublished
AmpC-104	ANW69906	383	−	−	Kulkarni *et al*., unpublished
AmpC-105	ANW69907	383	−	−	Kulkarni *et al*., unpublished
AmpC-106	ANW69909	383	−	−	Kulkarni *et al*., unpublished
AmpC-107	ANW69912	383	−	−	Kulkarni *et al*., unpublished
AmpC-108	AFI94770	383	WP_001211216	−	22952140
AmpC-109	AAV32519	383	−	−	16441449
AmpC-110	ABO38124	383	−	−	Huang *et al*., unpublished
AmpC-111	ABV21800	384	WP_001211220	−	18591275
AmpC-112	ABV21801	383	−	−	18591275
AmpC-113	ABV21802	383	−	−	18591275
**AmpC protein variant**	**GenBank accession number**	**Size (amino acid)**	**NCBI reference sequence**	**Other previous designations**	**PubMed IDentifier (PMID) / GenBank submission authors**
AmpC-114	ETY67158	384	−	−	20609238
AmpC-115	AFU38919	383	−	−	23209228
AmpC-116	WP_017816757	383	WP_017816757	−	23723398
AmpC-117	ELW88222	383	WP_002157727	−	Harkins *et al*., unpublished
AmpC-118	ENW75976	383	WP_001211227	ADC-CIP 70–34^T^	24277043
AmpC-119	ENU51112	383	WP_004712857	ADC-NIPH 1669	24277043
AmpC-120	ENV26641	383	WP_002126587	ADC-NIPH 190	24277043
AmpC-121	ENW36647	383	WP_005109685	ADC-NIPH 201	24277043
AmpC-122	ENW46489	383	WP_005123276	ADC-NIPH 329	24277043
AmpC-123	ENV30802	383	WP_004840559	ADC-NIPH 60	24277043
AmpC-124	ENW51893	383	WP_005128228	ADC-NIPH 601	24277043
AmpC-125	ENW51227	383	WP_005131186	ADC-NIPH 67	24277043
AmpC-126	ENW72863	383	WP_005138362	ADC-NIPH 80	24277043
AmpC-127	ENW00696	383	WP_005046018	ADC-CIP 81–8^T^	24277043
AmpC-128	ENU07956	383	WP_004643536	ADC-NIPH 13	24277043
AmpC-129	ENV92309	383	WP_005039111	ADC-ANC 3680	24277043
AmpC-130	ENV41121	383	WP_004886093	ADC-NIPH 386	24277043
AmpC-131	ENU48760	383	WP_004707701	ADC-NIPH 2119^T^	24277043
AmpC-132	ENW11417	383	WP_005068074	ADC-ANC 3678	24277043
AmpC-133	ENU43147	383	WP_004700205	ADC-NIPH 973	24277043
AmpC-134	ENX43770	383	WP_005307218	ADC-NIPH 542	24277043
AmpC-135	ENV03983	383	WP_004790939	ADC-NIPH 817	24277043
AmpC-136	EOQ64883	383	WP_016137488	ADC-ANC 3811	24277043
AmpC-137	EOQ71234	383	WP_016140427	ADC-ANC 4050	24277043
AmpC-138	EOQ73533	383	WP_016146025	ADC-ANC 4052	24277043
AmpC-139	EXS60093	383	WP_032039838	−	Harris *et al*., unpublished
AmpC-140	EYS55294	383	WP_001211209	−	Harris *et al*., unpublished
AmpC-141	EXD64655	383	WP_032062810	−	Harris *et al*., unpublished
AmpC-142	ETP95102	383	WP_031980335	−	24449752
AmpC-143	WP_033502167	383	WP_033502167	−	Liou *et al*., unpublished
AmpC-144	WP_001211214	383	WP_001211214	−	Sahl *et al*., unpublished
AmpC-145	KHY08585	383	WP_039270258	−	Adams *et al*., unpublished
AmpC-146	KHV30477	383	WP_039258389	−	Adams *et al*., unpublished
AmpC-147	KJC71195	383	WP_044718369	−	Adams *et al*., unpublished
AmpC-148	AJB47604	383	WP_039246976	−	McCorrison *et al*., unpublished
AmpC-149	ADY82440	383	WP_014207272	−	21441526
AmpC-150	AKT73351	383	WP_017386568	−	Ang *et al*., unpublished
AmpC-151	AMX20227	383	WP_063099318	−	Brasiliense *et al*., unpublished
AmpC-152	ADI89941	383	WP_013197184	−	20639327

^a^ AmpC-70 has the same amino acid sequence as AmpC-72 (ADC-72) with the GenBank accession number AIL90389 and PubMed IDentifier 25181293

## Concluding remarks

In our opinion, having two databases, one for the gene alleles and one for the protein variants, will create a lot of confusion. With the rapid accumulation of bacterial whole genome sequences, we argue that genes and alleles should reasonably be defined and numbered based on their nucleotide identities. For molecular epidemiological studies, the novel database for *ampC* in *A*. *baumannii* will provide unambiguous details beyond traditional list of AmpC variants that are limited to alleles with amino acid substitutions. To conclude, we emphasize on using the basic definition of the word “allele” for bacterial genes, by which novel alleles should be defined regardless if they are associated with amino acid changes or not.
